# The effect of healthcare professional-implemented interventions on adherence to oral targeted therapy in patients with cancer: a systematic review and meta-analysis

**DOI:** 10.1007/s00520-024-09136-4

**Published:** 2025-01-17

**Authors:** Fiona Angus, Wan-Chuen Liao, Victoria Adekoya, Li-Chia Chen

**Affiliations:** 1 Centre for Pharmacoepidemiology and Drug Safety, Division of Pharmacy and Optometry, Faculty of Biology, Medicine and Health, School of Health Sciences, Manchester Academic Health Science Centre, University of Manchester, Oxford Road, Manchester, M13 9PT UK; 2 Pharmacy Department, Christie NHS Foundation Trust, Manchester, UK; 3 College of Medicine, School of Dentistry, National Taiwan University, Taipei, Taiwan

**Keywords:** Healthcare professional, Interventions, Medication adherence, Oral targeted therapy, Cancer, Behavior change technique

## Abstract

**Purpose:**

This study investigated the impact of healthcare professional-led interventions on adherence to oral targeted therapy and identified the behavior change techniques (BCTs) underpinning the interventions.

**Methods:**

A systematic search of MEDLINE, Embase, APA PsycInfo, CINAHL Plus, PubMed, and Web of Science up to July 2024 identified randomized controlled trials and cohort studies involving adult patients (≥ 18 years) with cancer on oral targeted therapy receiving healthcare professional-led interventions to improve adherence. Adherence-related outcomes, including proportions of patients continuing treatments or with a medication possession ratio (MPR) ≥ 90%, were compared between intervention and control (usual care) groups. Pooled odds ratios (ORs) with 95% confidence intervals (CIs) and heterogeneity (*I*^2^ statistic) were reported. Differences in median time to treatment discontinuation were calculated and synthesized where applicable. Interventions were categorized using the BCT taxonomy.

**Results:**

This review included 11 studies (1,654 patients). The pooled results for proportions of patients continuing treatment (OR 17.91; 95%CI 3.18, 100.73; *I*^*2*^ < 0.1%) or with an MPR ≥ 90% (OR 3.67; 95%CI 1.98, 6.80; *I*^*2*^ < 0.1%) showed a significantly favorable outcome in the intervention group compared to the control group. In two studies, the median time to treatment discontinuation was longer in the intervention group than in the control group. The most commonly used BCTs were “credible source” (*n* = 11), “problem-solving” (*n* = 9), “instruction on how to perform a behavior” (*n* = 9), and “pharmacological support” (*n* = 8).

**Conclusion:**

Despite limited evidence, healthcare professional–led interventions significantly improve treatment adherence. Future studies should tailor strategies for individual needs and apply BCTs in designing effective interventions.

PROSPERO registered: no. CRD42024571808.

**Supplementary Information:**

The online version contains supplementary material available at 10.1007/s00520-024-09136-4.

## Introduction

Cancer remains a leading cause of morbidity and mortality worldwide, placing a significant burden on public health. In 2020, approximately 19.3 million new cases and nearly 10 million cancer-related deaths were reported globally [1], highlighting challenges not only in diagnosis and treatment but also in long-term survivorship and quality of life. Surgery, systemic anti-cancer therapy (SACT), and radiotherapy are the primary modalities for cancer treatment [2]. SACT describes all drugs used to treat cancer which includes chemotherapy, hormone therapy, targeted cancer drugs, and immunotherapy and may be used alone or in combination with surgery and radiation [2]. Despite chemotherapy being a vital treatment approach, the low drug concentrations in tumors, systemic toxicities, and a lack of specificity for tumor cells hindered its clinical treatment effectiveness [3]. The tumor responses can be short-lived, limited, and unpredictable [4].

In contrast, targeted therapies are designed to inhibit specific proteins, processes, and pathways that drive cancer cell growth. These are drugs therefore that treat cancer in a more precise way than chemotherapy [5] and have become available over the last 20 years or so as scientific knowledge in this area has advanced [6]. This approach has led to treatments with broader therapeutic windows and reduced toxicity [6]. Since cancers differ in their growth mechanisms — shaped by genetic mutations, environmental factors, and interactions with the immune system — targeted therapies are tailored accordingly, such as angiogenesis inhibitors for renal cell carcinoma and BRAF inhibitors for melanoma [7, 8]. These therapies generally cause fewer side effects than traditional chemotherapy, as they are more specific to cancer cells [9]. However, they can still cause mechanism-related toxicities, which require careful monitoring [10].

Increasingly, more cancer types are being treated with targeted therapies. As of 2020, 71 oral anti-cancer drugs targeting specific molecules have been approved, significantly improving cancer prognosis [11]. These therapies are typically self-administered oral agents used until they become intolerable or ineffective [12]. In contrast, traditional chemotherapy is usually administered intravenously over a set number of cycles and managed by healthcare professionals. Patients often prefer oral medications for their reduced discomfort and greater flexibility [12]. However, the long-term use of oral anti-cancer agents has raised concerns about medication adherence, which is crucial for ensuring adequate drug exposure, treatment efficacy, and long-term effectiveness [13]. Medication adherence can be defined as “the extent to which a person’s behaviour (including medication-taking) corresponds with agreed recommendations from a healthcare provider” and is affected by factors relating to the patient, disease, health system, therapy, and socioeconomic setting [14]. Suboptimal adherence has been associated with elevated medical expenses [15].

In addition to non-adherence, patients self-administering oral targeted therapies face challenges like polypharmacy, drug interactions, prescription errors, and unexpected side effects [16]. Adherence often declines over time; for instance, a study showed that only 59% of patients with hematologic malignancies remained on oral target therapy after 12 months [17]. Given the complexities of factors influencing [18], healthcare practitioners are crucial in educating patients, supporting adherence, and managing side effects [19]. As cancer treatment increasingly shifts toward oral therapies, developing strategies to address adherence and these associated challenges becomes essential [20].

Building on previous efforts to improve patient adherence through pharmaceutical support [16], collaboration with healthcare professionals [21], and the establishment of medical support teams [22] [23] to increase patients’ adherence and awareness, cognitive-based behavior change techniques (BCTs) have emerged as effective interventions to enhance patients’ self-efficacy, problem-solving, and motivation to improve adherence [24]. These techniques, categorized by BCT taxonomy including 93 hierarchically clustered methods [25], integrate behavioral and psychological approaches to address the connection between thoughts, feelings, and behaviors [26], which can help patients overcome negative patterns that hinder medication adherence. Hence, BCTs should be considered a framework when designing interventions. They have been applied in adherence improvement programs to increase compliance with oral anti-cancer therapies by addressing patients’ willingness and ability to change their behaviors [27].

While previous reviews have explored adherence [28], associated factors [29], and interventions in oral anti-cancer medications [30], research specifically concerning oral targeted therapies remains limited. The lack of data on healthcare professional-led interventions and their basis in behavior change theory highlights a significant knowledge gap. Mapping these interventions to the BCT taxonomy [25] is essential for understanding and improving patient adherence, enabling more tailored, patient-centered cancer treatment [31, 32], and informing future interventions for patients with complex conditions and diverse backgrounds. Therefore, this systematic review and meta-analysis aimed to evaluate the impact of healthcare professional-led interventions on adherence to oral targeted therapies in patients with cancer and identify BCTs aligning effective interventions.

## Methods

This systematic review and meta-analysis followed the Preferred Reporting Items for Systematic Reviews and Meta-Analyses (PRISMA) statement guidelines [33] (Appendix 1). The protocol has been registered at PROSPERO (no. CRD42024571808).

### Selection criteria

The inclusion and exclusion criteria of this study are summarized as follows (Table 1).

**Table 1:** Inclusion and exclusion criteria of this study

Component	Inclusion criteria	Exclusion criteria
Population and conditions	• Patients with cancer who are ≥ 18 years old and are receiving oral targeted therapy for cancer treatment	• Patients with cancer who are not taking oral targeted therapy • Patients with cancer include pediatrics, children, adolescents, neonates or infants
Intervention and comparator	• Healthcare professionals delivered interventions to be received by patients aiming to improve adherence • The comparison group comprises patients with cancer taking oral targeted therapy and receiving standard/usual care without healthcare professionals delivering additional interventions to improve adherence	• Medical or surgical interventions, including invasive procedures and transplantation-based treatments • Family/group-based interventions
Outcome	• Report medication adherence outcomes for the intervention and control groups or the effect size of adherence between the two groups	• No report on medication adherence
Study type	Human studies	Animal or in vitro studies
Language	English	Other languages without English translation
Publication	Randomized controlled trials and cohort studies	Qualitative studies, case reports, case series, reviews, systematic reviews, case–control studies, descriptive cross-sectional studies, study protocols, feasibility or pilot studies, editorials, conference abstracts, and grey literature

### Types of studies

Randomized controlled trials and cohort studies were included. Qualitative studies, case reports, case series, reviews, systematic reviews, case–control studies, descriptive cross-sectional studies, study protocols, feasibility or pilot studies, editorials, conference abstracts, and gray literature were excluded.

### Types of participants

The study included participants who are patients with cancer, aged 18 years and older, and receiving oral targeted therapy. Patients with cancer who are not taking oral targeted therapy, as well as studies involving pediatrics, children, adolescents, neonates, or infants, were excluded.

### Types of interventions

Healthcare professionals who delivered interventions aimed at improving adherence in patients were included. Previous literature [34] and the Cochrane Effective Practice and Organisation of Care (EPOC) taxonomy of health system interventions [35] facilitated the definition of delivered interventions. In addition, the research team identified categories and items from the EPOC taxonomy that are directly relevant to the interventions (Appendix 2) and considered the inclusion criteria. Other types of involvement, such as medical or surgical interventions, including invasive procedures, transplantation-based treatments, or family or group-based interventions, were excluded.

### Types of outcome measures

Oral targeted therapy adherence is the outcome measure in this study. Medication adherence outcomes were included, such as adherence rate, medication possession ratio, number of missed doses, correct medication intake, time to treatment discontinuation, and the effect size of adherence between the intervention and control groups. Studies without reporting medication adherence results were excluded.

### Data sources and search strategies

A comprehensive electronic database search was conducted on the MEDLINE, Embase, APA PsycInfo, CINAHL Plus, PubMed, and Web of Science databases from inception to July 2024. Various structured search strategies were employed using controlled vocabulary and keywords based on this study’s inclusion and exclusion criteria (Table 1) (Appendix 3). The keywords and terms of interventions and outcomes were guided by the EPOC taxonomy and a systematic review by Tan et al. (2021) [34]. Restrictions were also applied to the search, including articles published in English and human studies.

### Study selection

The title and abstract of articles retrieved from the electronic databases search were first screened by two reviewers (FA and WCL) independently according to the selection criteria (Table 1) using the pre-designed electronic screening form. Each article was rated as either “included,” “further check,” or “excluded.” The intraclass correlation coefficient (two-way mixed effect model with absolute agreement) [36] and 95% confidence interval (95%CI) were calculated for the consistency between two reviewers (FA and WCL) in record screening. Any discrepancy was resolved by discussing between reviewers and, if necessary, with a third reviewer (LCC) to reach a consensus. The full texts of potentially eligible articles were further reviewed independently by two reviewers (FA and WCL) to conclude the selection of studies.

### Data extraction and management

The data extraction process for each study will be conducted independently by two reviewers (FA and WCL) using the standardized and piloted electronic data extraction sheet. A third reviewer (LCC) will adjudicate disagreements. The following data will be extracted from each included study and presented as follows: (1) study information: study title, leading author, country, and year of publication; (2) study design: setting, targeted population (cancer type, which treatment they were on, and for how long), intervention (type of intervention), comparison, adherence outcome measures, duration, and statistical analysis; (3) results: continuous data (such as median time to treatment discontinuation and 95%CI) and dichotomous data (such as proportion of adherence rate).

### Risk of bias assessment

Quality assessment of all included studies was conducted using the Cochrane Risk of Bias Assessment Tool (RoB 2) for randomized controlled trials and the Risk of Bias in Non-randomized Studies of Interventions tool (ROBINS-I) for non-randomized studies [37, 38]. The articles were classified into low risk of bias, some concerns, or high risk of bias in RoB 2, and low, moderate, serious, or critical risk of bias in ROBINS-I. The results were tabulated.

### Data analysis and presentation

The outcomes were compared between the intervention group (those receiving healthcare professional-implemented interventions) and the control group (those receiving standard care only or no intervention). The proportions of adherence results were synthesized using a random-effect model (Der-Simonian and Laird method [39]). Pooled effect sizes were reported using odds ratios (ORs) and 95%CIs, with heterogeneity assessed using the *I*^*2*^ test (%). The difference in median time to treatment discontinuation (by subtraction) was calculated and synthesized where appropriate. The interventions described in the included studies, aimed at improving adherence to oral targeted therapy, were selected and classified using the BCT taxonomy by two reviewers (FA and WCL). This taxonomy consists of 93 techniques organized into 16 hierarchical clusters. The techniques used in each study were then tabulated [25]. Meta-analysis was conducted using STATA (Release 14. College Station, TX: StataCorp LLC).

## Results

### Selection of study

**Fig. 1:**
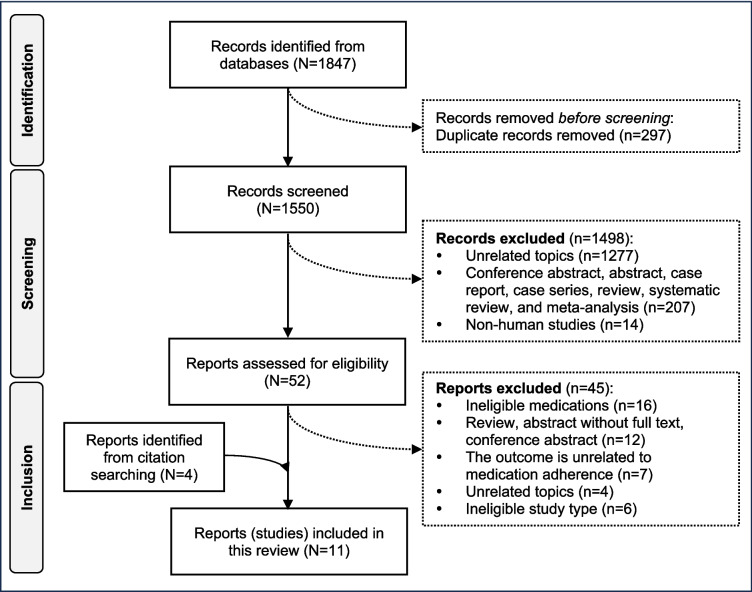
Selection of studies.

A total of 1847 records were identified through electronic database searches. After removing 297 duplicates and 1498 irrelevant records, including studies non-relevant to healthcare professional-implemented interventions to improve adherence to oral targeted therapy (*n* = 1277), abstracts without full text, case reports, reviews (*n* = 207), and non-human studies (*n* = 14). Following the full-text screening of the remaining 52 articles, 45 were excluded, resulting in seven studies. Additionally, four articles were identified through citation searching, resulting in 11 studies (1654 patients) included in this review (Fig. 1). The intraclass correlation coefficient between reviewers was 0.933 (95%CI 0.923, 0.941), indicating good consistency.

### Characteristics of study

Eleven studies, including four randomized controlled trials [27, 40–42] and seven cohort studies [21–23, 43–46] (Table 2), were conducted in the USA (*n* = 3) [43, 44, 46], Japan (*n* = 3) [21–23], Switzerland (*n* = 2) [27, 42], and once each in Finland [40], Malaysia [41], and France [45]. Studies involved patients with chronic myeloid leukemia (*n* = 4) [40, 41, 44, 46], renal cell carcinoma (*n* = 2) [22, 23], breast cancer (*n* = 1) [42], and various cancer types (*n* = 4) [21, 27, 43, 45]. Gender distribution was not balanced in nine studies [21–23, 27, 40, 41, 44–46], and two studies [42, 43] did not report gender data. Medications included pazopanib (*n* = 2) [22, 23], sorafenib (*n* = 1) [21], ibrutinib (*n* = 1) [45], palbociclib (*n* = 1) [42], and other targeted therapy medications (*n* = 6) [27, 40, 41, 43, 44, 46]. The 11 studies included 741 patients in the intervention group and 913 in the control group. Outcome measures reported were medication possession ratio (*n* = 4) [41, 43–45], number of patients who discontinued treatment (*n* = 3) [21–23], duration of treatment (*n* = 2) [22, 23], medication adherence scale (*n* = 2) [40, 46], number of patients missing at least one dose (*n* = 1) [42], and proportions of patients with correct medication intake (*n* = 1) [27].

**Table 2:** Characteristics of included studies

Author, year, country	Study design, types of cancer, number of patients	Medication	Age (years), gender	Intervention	Control	Outcome measure
Khandelwal, 2012, US [43]	Cohort study Various cancer types^*^ (*N* = 754)	Sorafenib, sunitinib, or erlotinib	Age not specified Gender not specified	Cycle management program (*n* = 377)	Without the program (*n* = 377)	MPR
Kajizono, 2015, Japan [21]	Cohort study Hepatocellular and advanced renal cell carcinoma (*N* = 70)	Sorafenib	Range: int. (51, 86) vs.; Con. (28, 88) Both groups: > 50% male	Medical supportive team (*n* = 31)	Without a supportive team (*n* = 39)	Number of patients who discontinued sorafenib
Kekale, 2016, Finland [40]	RCT Chronic myeloid leukemia (*N* = 68)	Imatinib, dasatinib, or nilotinib	Median (range): 60 (25–83) Int. > 50% female vs. Con. > 50% male	Nurse-led medication counselling, an information booklet, video and website, and text message reminders (= 35)	Standard treatment (*n* = 33)	Morisky Medical Adherence Scale
Lam, 2016, US [44]	Cohort study Chronic myelogenous leukemia (*N* = 269)	Imatinib, dasatinib, nilotinib, bosutinib, or ponatinib	Median (range): int. 57 (29–83) vs. Con. 54.9 (18.4–92.8) Both groups: > 50% male	Pharmacist monitoring (*n* = 44)	Usual care (*n* = 225)	MPR
Todo, 2019, Japan [23]	Cohort study Metastatic renal cell carcinoma (*N* = 50)	Pazopanib	Median (range): Int. 67 (46–80) vs Con. 66 (43–80) Both groups: > 50% male	Patient-pharmacist interviews and a hotline (*n* = 37)	Without interventions (*n* = 13)	Duration of treatment and cases of discontinuation
Tan, 2020, Malaysia [41]	RCT Chronic myeloid leukemia (*N* = 129)	Imatinib, nilotinib, dasatinib, or ponatinib	Median (IQR): Int. 44.5 (32,56) vs. Con. 40.5 (33,55) Both groups: > 50% male	Medication management service (*n* = 35)	Usual care (*n* = 64)	MPR
Zerbit, 2020, France [45]	Cohort study Various cancer types^†^ (*N* = 68)	Ibrutinib	Median (IQR): Int. 66 (59.3, 73.4) vs. Con: 69 (62.0, 77.5) Both groups: > 50% male	Pharmaceutical care program (*n* = 34)	Usual care (*n* = 34)	MPR
Dennison, 2021, US [46]	Cohort study Chronic myeloid leukemia (*N* = 40)	Imatinib, dasatinib, bosutinib, or nilotinib	Mean (± SD): Int. 57.35 ± 13.97 vs. Con. 53.25 ± 11.84 Int. > 50% male vs. Con. > 50% female	Pharmacist follow-up (*n* = 20)	Standard care (*n* = 20)	Morisky Green Levine Medication Adherence Scale
Hirabatake, 2023, Japan [22]	Cohort study Renal cell carcinoma (*N* = 51)	Pazopanib	Median (IQR): 69 (64–76) vs. Con. 71 (65–76) Both groups: > 50% male	Pharmacist-urologist collaborative management (*n* = 21)	Without collaboration approach (*n* = 30)	Duration of treatment and cases of discontinuation
Bandiera, 2023, Switzerland [42]	RCT Metastatic breast cancer (*N* = 37)	Palbociclib	Median (IQR): 62 (52, 73) vs. Con. 64 (55, 75) Gender not specified	Interprofessional medication adherence program (*n* = 19)	Without the program (*n* = 18)	Number of patients without missing dose
Bandiera, 2024, Switzerland [27]	RCT Solid cancers (*N* = 118)	Various medications^§^	Median (IQR): Int. 61 (53.0, 70.7) vs. Con. (52.2, 68.4) Both groups: > 50% female	Patient-pharmacist interview (*n* = 58)	Standard care	Proportion of patients with correct medication intake

(Note) *US*, USA; *RCT*, randomized controlled trial. ^*^Liver or kidney cancers; gastrointestinal stromal tumors; or non-small cell lung and pancreatic cancers. ^†^Chronic lymphocytic leukemia, Waldenström macroglobulinaemia, mantle cell lymphoma, or other B cell malignancies. ^§^Palbociclib, regorafenib, pazopanib, imatinib, sorafenib, everolimus, lenvatinib, trametinib/dabrafenib, axitinib, olaparib, cobimetinib, erdafitinib, erlotinib, niraparib, trametinib, trametinib/olaparib, vemurafenib, cabozantinib, binimetinib, alectinib, cobimetinib/vemurafenib, encorafenib, lapatinib, osimertinib, ribociclib, sunitinib, or neratinib. *IQR*, interquartile range; *SD*, standard deviation; *MPR*, medication possession ratio; *Int.*, intervention grou; *Con.*, control group

### Characteristics of interventions

Most interventions took place in hospitals, medical centers, and oncology clinics (*n* = 10) [21–23, 27, 40–42, 44–46], with one at a specialty pharmacy [43]. Patients primarily self-administered oral targeted therapy (*n* = 9) [21–23, 40, 41, 43–46]. Two studies used a medication event monitoring system with a pill bottle cap sensor to track drug intake [27, 42]. The interventions were mainly delivered by pharmacists (*n* = 8) [22, 23, 27, 41, 42, 44–46], with two involving both pharmacists and nurses [21, 43] and one by nurses alone [40] (Appendix 4).

The interventions were categorized into four strategies: side effect management (*n* = 11) [21–23, 27, 40–46], adherence monitoring (*n* = 10) [22, 23, 27, 40–46], disease and medication education (*n* = 9) [23, 27, 40–46] and drug interaction reviews [22, 41, 44–46]. Some specific approaches for improving adherence included daily text reminders (*n* = 1) [40], medication packs with dates and a reminder application (*n* = 1) [41], health care professional-led caregiver training (*n* = 1) [45], scheduled visits every 2–4 weeks (*n* = 1) [46], and investigating willingness of behavior change (*n* = 1) [27]. Coordination between healthcare teams and advising physicians on adherence, side effects, and drug interactions was noted in seven studies [21–23, 27, 42, 43, 45] (Appendix 4).

Most interventions were delivered face-to-face (*n* = 8) [21–23, 27, 42, 44–46], with some using multimedia interactive information tools (*n* = 1) [40] or follow-up calls (*n* = 1) [41], and one by telephone [43]. Intervention frequency varied from weekly or monthly (*n* = 4) [21, 27, 42, 43] to every 3–6 months (*n* = 1) [45], with various intervals between interventions (*n* = 3) [41, 44, 46] or unspecified frequency (*n* = 3) [22, 23, 40]. The longest intervention duration was 6 years (*n* = 1) [44], with others spanning 37–60 months (*n* = 2) [21, 22], 25–36 months (*n* = 1) [23], 13–24 months (*n* = 1) [45], and 3–12 months (*n* = 6) [27, 40–43, 46] (Appendix 4).

### Quality assessment

The four randomized controlled trials raised concerns about contamination bias in the control group [27, 40–42]. Issues included randomization conducted by the main investigator [40] and non-randomized in the control group [42]. Two studies also had concerns about bias from missing outcome data [27, 40] and potential measurement bias for patient self-reports [40] (Appendix 5).

The seven cohort studies had moderate concerns due to confounding bias from various cancers and targeted therapies [21–23, 43–46]. Patient self-reported outcomes may introduce measurement bias [46]. One study exclusively analyzed patients on imatinib, which may have biased results by selected reporting [44] (Appendix 6).

### Outcome measurement

Pooled results indicated that the intervention group had significantly better outcomes compared to the control group for both the proportions of patients continuing treatment (*n* = 3; OR 17.91; 95%CI 3.18, 100.73; *I*^*2*^ < 0.1%) and medication possession ratio ≥ 90% (*n* = 2; OR 3.67; 95%CI 1.98, 6.80; *I*^*2*^ < 0.1%). However, the 90%CI for the proportion of patients continuing treatment was very wide. There was no significant difference but heterogenous in the proportion of patient-reported high adherence (*n* = 2; OR 1.86; 95%CI 0.40, 8.67; *I*^*2*^ = 71.5%). Additionally, no significant differences were found for the proportion of patients without missing doses (*n* = 1; OR 4.50; 95%CI 0.97, 20.83) or with correct medication intake (*n* = 1; OR 1.33; 95%CI 0.43, 4.11) between the intervention and control groups (Table 3). The mean MPR was higher in the intervention group than in the control group in the two studies, at 44.8% vs. 41.5% [43] and 99% vs. 90% [45]. Two studies reported that the intervention group had a longer median treatment discontinuation time than the control group (Table 3).

**Table 3:**
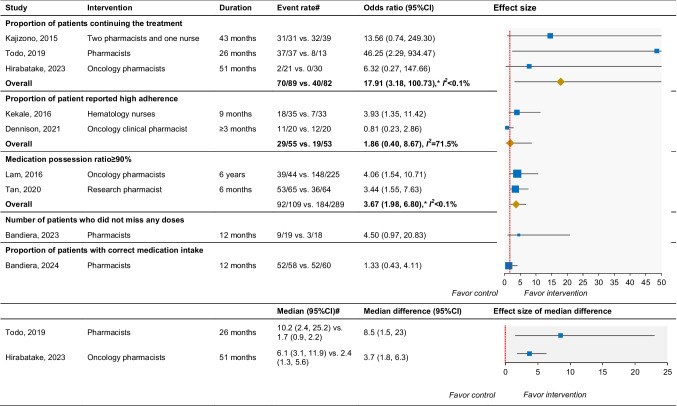
Adherence outcome

(Note) # Intervention group vs. control group. CI: confidence interval. * Significant difference

### Behavior change techniques applied in interventions

A total of 20 out of 93 BCTs were identified across the included studies, with each study applying between 5 and 13 BCTs. The most commonly used techniques were “credible source” (*n* = 11) [21–23, 27, 40–46], “problem-solving” (*n* = 9) [21, 23, 27, 40–43, 45, 46], “instruction on how to perform a behavior” (*n* = 9) [23, 27, 40–46], and “pharmacological support” (*n* = 8) [21–23, 41, 42, 44–46]. The least used techniques included “goal setting (behavior)” (*n* = 1) [45], “discrepancy between current behavior and goal” (*n* = 1) [42], “monitoring of behavior by others without feedback” (*n* = 1) [21], and “social support (practical)” (*n* = 1) [43] (Table 4).

**Table 4:** Behavior change techniques applied in the eleven included studies

Study and the interventions		Group and BCTs																				
		1. Goals and planning							2. Feedback and monitoring						3. Social support		4. Shaping knowledge	5. Natural consequences	7. Associations	9. Comparison of outcomes	11. Regulation	Number of BCTs applied
		1.1	1.2	1.3	1.4	1.5	1.6	1.7	2.1	2.2	2.3	2.4	2.5	2.7	3.1	3.2	4.1	5.1	7.1	9.1	11.1	
Khandelwal, 2012 [43]	Cycle management program		V			V		V		V		V			V	V	V	V		V		10
Kajizono, 2015 [21]	Medical supportive team		V						V				V							V	V	5
Kekale, 2016 [40]	Nurse-led medication counselling, an information booklet, video and website, and text message reminders		V	V	V							V			V		V	V	V	V		9
Lam, 2016 [44]	Pharmacist monitoring					V							V				V	V		V	V	6
Todo, 2019 [23]	Patient-pharmacist interviews and a hotline		V		V	V		V			V	V			V		V			V	V	10
Tan, 2020 [41]	Medication management service		V	V	V	V									V		V	V	V	V	V	10
Zerbit, 2020 [45]	Pharmaceutical care program	V	V		V			V			V	V	V	V	V		V			V	V	12
Dennison, 2021 (46)	Pharmacist follow-up		V		V			V						V	V		V	V		V	V	9
Hirabatake, 2023 [22]	Pharmacist-urologist collaborative management							V			V		V							V	V	5
Bandiera, 2023 [42]	Interprofessional medication adherence program		V		V	V	V	V		V		V	V				V	V	V	V	V	13
Bandiera, 2024 [27]	Patient-pharmacist interview		V		V	V		V		V	V	V	V	V			V	V		V		12
**Frequency of BCTs identified**		1	9	2	7	6	1	7	1	3	4	6	6	3	6	1	9	7	3	11	8	101

(Note) *BCT*, behavior change technique. 1.1 Goal setting (behavior). 1.2 Problem-solving. 1.3 Goal setting (outcome). 1.4 Action planning. 1.5 Review behavior goal(s). 1.6 Discrepancy between current behavior and goal. 1.7 Review outcome goal(s). 2.1 Monitoring behavior by others without feedback. 2.2 Feedback on behavior. 2.3 Self-monitoring of behavior. 2.4 Self-monitoring of outcome(s) of behavior. 2.5 Monitoring of outcome(s) of behavior without feedback. 2.7 Feedback on outcome(s) of behavior. 3.1 Social support (unspecified). 3.2 Social support (practical). 4.1 Instruction on how to perform a behavior. 5.1 Information about health consequences. 7.1 Prompts/cues. 9.1 Credible source. 11.1 Pharmacological support

## Discussion

This review found most studies indicated better adherence outcomes in the intervention groups, although not all differences were statistically significant. Despite a wide confidence interval for treatment continuity, there were significant improvements in the proportions of treatment continuation and MPR ≥ 90% in the intervention group. Most of the interventions in the included studies involve discussing side effect management, adherence monitoring, disease and medication education, and some also include notifications for patients. Pharmacists played a crucial role in delivering these interventions. The most commonly used BCTs were “credible source,” “problem-solving,” “instruction on how to perform a behavior,” and “pharmacological support”.

This review identified various strategies healthcare professionals employ to enhance adherence to oral targeted therapy. Unlike studies focusing solely on digital intervention [47, 48], healthcare professionals, particularly pharmacists, served as a “credible source” for patients, supporting them with “problem-solving,” “instruction on behavior,” and “pharmacological support” — the most frequently used BCTs in this review. Survey data further emphasize the importance of pharmacists in cancer care, with 86% of patients valuing treatment discussions and 76% seeking follow-up with a pharmacist [49].

Given the complexity of medication adherence, educational materials alone may not significantly impact outcomes [50]. Common barriers to adherence, such as side effects, forgetfulness, and lack of prescription knowledge [51], require targeted behavioral interventions. Effective strategies include providing information on adverse events, setting reminders, and clarifying therapy goals [44]. Proper management of side effects is crucial for preventing non-compliance [52], with many studies emphasizing patient education on self-managing these issues [23, 27, 40–42, 45, 46]. The “problem-solving” BCT was frequently used to address the causes of non-adherence and manage drug-related problems [21, 23, 27, 40–43, 45, 46], while “pharmacological support” helped patients anticipate and manage drug-drug interactions and necessary medication adjustments.

A previous systematic review by Pezzolato et al. (2023) on interventions to improve adherence to oral anti-cancer medication in patients with breast cancer identified the “feedback on behavior” technique as significantly effective in enhancing adherence rates [32]. However, our review found only three studies applied this technique [27, 42, 43]. This suggests that different BCTs can be beneficial, highlighting the importance of understanding individual behaviors and tailoring treatment strategies accordingly.

Our systematic review is the first to specifically examine healthcare professional interventions aimed at improving adherence to oral targeted therapy and to map these interventions using the BCT taxonomy. By synthesizing adherence-related data from the included studies, we provided novel pooled results not previously reported. These findings underscore the critical role of healthcare providers and the potential benefits of integrating behavioral and psychological approaches.

However, several limitations must be acknowledged. The observed heterogeneity and wide 95%CI in effect size are due to inconsistencies in adherence measures and variations in patient characteristics, cancer types and stages, targeted therapies, side effects, comorbidities, polypharmacy, medications, and the types of intervention. Potential contamination bias was present in the control groups of the randomized controlled trials [41]. In observational cohort studies, confounding factors such as varying cancer types, medications, and healthcare interventions introduced unavoidable bias. Variability in pharmaceutical programs, the content and intensity of information provided, intervention duration, and gender imbalances also potentially influenced outcomes. Additionally, the results are specific to the types of oral targeted therapies included, which may limit their generalizability.

Most studies do not provide a distinct definition for adherence [22–24, 45–47] although they typically outline the outcome measures used to assess adherence within their respective contexts. Three studies conceptualized adherence as a behavior related to “taking a medication as prescribed,” aligning with the World Health Organization’s definition [28, 41, 44]. Among these, Bandiera et al. (2024) further characterize adherence through three phases: initiation, implementation, and discontinuation [28]. Another study by the same authors supported this tripartite framework [43]. This definition significantly influences the outcome measures employed, as both studies specifically calculate adherence during the implementation phase, with the former also incorporating a secondary measure that examines the duration from initiation to discontinuation. It is crucial to recognize that differing definitions of adherence, despite not always being explicitly stated, can lead to variations in the outcome measures. Consequently, these discrepancies may reflect the authors’ individual interpretations of adherence.

Adherence to oral anti-cancer medication can be measured through direct (e.g., serum drug levels) and indirect methods (e.g., pill counts, electronic tracking, refill records, self-reports) [53], but a reliable and clinically relevant scale specifically for oral targeted therapy is still lacking [54], and inconsistencies in adherence monitoring methods persist [55]. As the availability of routinely collected data and patient-reported outcome measures in real-world settings increases, future research should focus on evaluating and validating adherence measures and outcomes for patients on oral anti-cancer therapies.

In addition, to optimize the use of oral targeted therapy, it is crucial that future interventions incorporate BCTs and actively involve patients in the co-design process to identify the most effective strategies for enhancing adherence. Qualitative studies exploring patient experiences with BCTs and the role of healthcare providers in real-world settings can provide valuable insights that complement quantitative data. Such research will support the development of tailored, patient-centered cancer treatment approaches that balance medication adherence with optimal treatment outcomes.

This systematic review outlined the current evidence on the effectiveness of healthcare professional-led interventions to improve adherence to oral targeted therapy in cancer patients. Additionally, the control group showed a wide range of adherence rates, consistent with previous reports of adherence to oral anti-cancer treatment ranging from less than 20 to 100% [53]; hence, there is still a need to address the barriers to adherence issues [52].

The findings of this review indicate that healthcare professional-led interventions can effectively improve adherence to oral targeted therapy, as evidenced by increased treatment continuation and medication possession ratio. Incorporating BCTs into these interventions appears to be promising. Integrating healthcare professional-led or medical support teams into routine treatment procedures for oral targeted therapy in cancer patients is advisable. Previously, BCTs have not been widely applied or reported for healthcare professional interventions, so the application in this study provides a good framework for understanding BCTs underpinning this intervention.

Interventions which include “credible sources,” “problem-solving,” “instruction on how to perform a behavior,” and “pharmacological support” have been shown to improve adherence and treatment outcomes for cancer patients taking oral targeted therapy. To further reduce treatment discontinuation risk and improve medication possession ratios in patients undergoing oral targeted therapy, future research should focus on validating the outcome measures and refining these intervention strategies by exploring and applying the BCTs identified in this study and investigating other potential BCTs.

## Supplementary Information

Below is the link to the electronic supplementary material.

Supplementary file1 (DOCX 88 KB)

## Data Availability

Research data is available upon request from the lead author.

## References

[1] Sung H, Ferlay J, Siegel RL, Laversanne M, Soerjomataram I, Jemal A et al (2021) Global cancer statistics 2020: GLOBOCAN estimates of incidence and mortality worldwide for 36 cancers in 185 countries. CA Cancer J Clin 71(3):209–249 33538338 10.3322/caac.21660

[2] Cancer Research UK (2024) Chemotherapy safety at home. Available from https://www.cancerresearchuk.org/about-cancer/treatment/chemotherapy/chemotherapy-safety-at-home. Accessed 30 June 2024

[3] Xu G, McLeod HL (2001) Strategies for enzyme/prodrug cancer therapy. Clin Cancer Res 7(11):3314–3324 11705842

[4] Arora A, Scholar EM (2005) Role of tyrosine kinase inhibitors in cancer therapy. J Pharmacol Exp Ther 315(3):971–979 16002463 10.1124/jpet.105.084145

[5] Sledge GW (2005) What is targeted therapy? J Clin Oncol 23(8):1614–1615 15755966 10.1200/JCO.2005.01.016

[6] Vickers E (2018) A Beginner's Guide to Targeted Cancer Treatments. Newark, United Kingdom: John Wiley & Sons, Incorporated

[7] Wooster R, Davies H, Bignell GR, Cox C, Stephens P, Edkins S et al (2002) Mutations of the BRAF gene in human cancer. Nature (London) 417(6892):949–954 12068308 10.1038/nature00766

[8] Ferrara N (2004) Vascular endothelial growth factor as a target for anticancer therapy. Oncologist 9(1):2–10 15178810 10.1634/theoncologist.9-suppl_1-2

[9] Carrington C (2015) Oral targeted therapy for cancer. Aust Prescr 38(5):171–176 26648656 10.18773/austprescr.2015.060 PMC4657306

[10] Shyam Sunder S, Sharma UC, Pokharel S (2023) Adverse effects of tyrosine kinase inhibitors in cancer therapy: pathophysiology, mechanisms and clinical management. Signal Transduct Target Ther 8(1):262 37414756 10.1038/s41392-023-01469-6 PMC10326056

[11] Mueller-Schoell A, Groenland SL, Scherf-Clavel O, van Dyk M, Huisinga W, Michelet R et al (2021) Therapeutic drug monitoring of oral targeted antineoplastic drugs. Eur J Clin Pharmacol 77(4):441–464 33165648 10.1007/s00228-020-03014-8 PMC7935845

[12] Eek D, Krohe M, Mazar I, Horsfield A, Pompilus F, Friebe R et al (2016) Patient-reported preferences for oral versus intravenous administration for the treatment of cancer: a review of the literature. Patient Prefer Adherence 10:1609–1621 10.2147/PPA.S106629 PMC5003561 27601886

[13] Barthélémy P, Asmane-De la Porte I, Meyer N, Duclos B, Serra S, Dourthe L-M et al (2014) Adherence and patients’ attitudes to oral anticancer drugs: a prospective series of 201 patients focusing on targeted therapies. Oncol 88(1):1–8 10.1159/000366226 25247774

[14] World Health Organisation (2003) Adherence to long-term therapies: evidence for action. Geneva World Health Organization. Available from: https://who.int/iris/handle/10665/42682. Accessed 9 Dec 2024

[15] Hall AE, Paul C, Bryant J, Lynagh MC, Rowlings P, Enjeti A et al (2016) To adhere or not to adhere: rates and reasons of medication adherence in hematological cancer patients. Crit Rev Oncol Hematol 97:247–262 26412718 10.1016/j.critrevonc.2015.08.025

[16] Dürr P, Schlichtig K, Kelz C, Deutsch B, Maas R, Eckart MJ et al (2021) The randomized AMBORA trial: impact of pharmacological/pharmaceutical care on medication safety and patient-reported outcomes during treatment with new oral anticancer agents. J Clin Oncol 39(18):1983–1994 33822650 10.1200/JCO.20.03088

[17] Patel K, Sudhir VS, Kabadi S, Huang JC, Porwal S, Thakkar K et al (2019) Impact of dosing frequency (once daily or twice daily) on patient adherence to oral targeted therapies for hematologic malignancies: a retrospective cohort study among managed care enrollees. J Oncol Pharm Pract 25(8):1897–1906 30823852 10.1177/1078155219827637 PMC6839022

[18] Verbrugghe M, Verhaeghe S, Lauwaert K, Beeckman D, Van Hecke A (2013) Determinants and associated factors influencing medication adherence and persistence to oral anticancer drugs: a systematic review. Cancer Treat Rev 39(6):610–621 23428230 10.1016/j.ctrv.2012.12.014

[19] Viele CS (2007) Managing oral chemotherapy: the healthcare practitioner’s role. Am J Health Syst Pharm. 64(9_Supplement_5):S25–S32 17468153 10.2146/ajhp070037

[20] Wood L (2012) A review on adherence management in patients on oral cancer therapies. Eur J Oncol Nurs 16(4):432–438 22051845 10.1016/j.ejon.2011.10.002

[21] Kajizono M, Aoyagi M, Kitamura Y, Sendo T (2015) Effectiveness of medical supportive team for outpatients treated with sorafenib: a retrospective study. J Pharm Health Care Sci 1:1–6 26819717 10.1186/s40780-014-0005-0 PMC4677728

[22] Hirabatake M, Ikesue H, Yoshino S, Morimoto M, Yamasaki T, Hashida T et al (2023) Pharmacist-urologist collaborative management for patients with renal cell carcinoma receiving pazopanib monotherapy. Biol Pharm Bull 46(8):1065–1071 37532558 10.1248/bpb.b22-00917

[23] Todo M, Shirotake S, Nishimoto K, Yasumizu Y, Kaneko G, Kondo H et al (2019) Usefulness of implementing comprehensive pharmaceutical care for metastatic renal cell carcinoma outpatients treated with pazopanib. Anticancer Res 39(2):999–1004 30711987 10.21873/anticanres.13205

[24] Easthall C, Song F, Bhattacharya D (2013) A meta-analysis of cognitive-based behaviour change techniques as interventions to improve medication adherence. BMJ Open 3(8):e002749 23935093 10.1136/bmjopen-2013-002749 PMC3740257

[25] Michie S, Richardson M, Johnston M, Abraham C, Francis J, Hardeman W et al (2013) The behavior change technique taxonomy (v1) of 93 hierarchically clustered techniques: building an international consensus for the reporting of behavior change interventions. Ann Behav Med 46(1):81–95 23512568 10.1007/s12160-013-9486-6

[26] Spoelstra SL, Schueller M, Hilton M, Ridenour K (2015) Interventions combining motivational interviewing and cognitive behaviour to promote medication adherence: a literature review. J Clin Nurs 24(9–10):1163–1173 25420723 10.1111/jocn.12738

[27] Bandiera C, Cardoso E, Locatelli I, Zaman K, Diciolla A, Digklia A et al (2024) A pharmacist-led interprofessional medication adherence program improved adherence to oral anticancer therapies: the OpTAT randomized controlled trial. PLoS ONE. 19:e0304573 38848380 10.1371/journal.pone.0304573 PMC11161104

[28] Greer JA, Amoyal N, Nisotel L, Fishbein JN, MacDonald J, Stagl J et al (2016) A systematic review of adherence to oral antineoplastic therapies. Oncologist 21(3):354–376 26921292 10.1634/theoncologist.2015-0405 PMC4786357

[29] Mathes T, Pieper D, Antoine SL, Eikermann M (2014) Adherence influencing factors in patients taking oral anticancer agents: a systematic review. Cancer Epidemiol 38(3):214–226 24768601 10.1016/j.canep.2014.03.012

[30] Mathes T, Antoine SL, Pieper D, Eikermann M (2014) Adherence enhancing interventions for oral anticancer agents: a systematic review. Cancer Treat Rev 40(1):102–108 23910455 10.1016/j.ctrv.2013.07.004

[31] Govender R, Smith CH, Taylor SA, Barratt H, Gardner B (2017) Swallowing interventions for the treatment of dysphagia after head and neck cancer: a systematic review of behavioural strategies used to promote patient adherence to swallowing exercises. BMC Cancer 17:1–15 28068939 10.1186/s12885-016-2990-x PMC5223405

[32] Pezzolato M, Marzorati C, Lanzoni L, Monzani D, Masiero MA, Pietrobon R et al (2023) Interventions to increase adherence to oral therapies in breast cancer patients: a systematic review based on the behavior change technique taxonomy. Psychooncology 32(10):1481–1502 37571974 10.1002/pon.6203

[33] Liberati A, Altman DG, Tetzlaff J, Mulrow C, Gøtzsche PC, Ioannidis JP, et al (2009) The PRISMA statement for reporting systematic reviews and meta-analyses of studies that evaluate health care interventions: explanation and elaboration. Ann Intern Med 151(4):W-65-W-94 10.7326/0003-4819-151-4-200908180-00136 19622512

[34] Tan BK, Bee PC, Chua SS, Chen LC (2021) Monitoring and improving adherence to tyrosine kinase inhibitors in patients with chronic myeloid leukemia: a systematic review. Patient Prefer Adherence 15:2563–2575 10.2147/PPA.S269355 PMC8608409 34819724

[35] Effective Practice and Organisation of Care (EPOC) (2015) EPOC Taxonomy. Available from: https://epoc.cochrane.org/epoc-taxonomy. Accessed 20 Apr 2024

[36] Koo TK, Li MY (2016) A guideline of selecting and reporting intraclass correlation coefficients for reliability research. J Chiropr Med 15(2):155–163 27330520 10.1016/j.jcm.2016.02.012 PMC4913118

[37] Sterne JA, Hernán MA, Reeves BC, Savović J, Berkman ND, Viswanathan M et al (2016) ROBINS-I: a tool for assessing risk of bias in non-randomised studies of interventions. BMJ 355:i4919 27733354 10.1136/bmj.i4919 PMC5062054

[38] Sterne JAC, Savović J, Page MJ, Elbers RG, Blencowe NS, Boutron I et al (2019) RoB 2: a revised tool for assessing risk of bias in randomised trials. BMJ 366:l4898 31462531 10.1136/bmj.l4898

[39] Kontopantelis E, Reeves D (2010) Metaan: random-effects meta-analysis. Stata J 10(3):395–407

[40] Kekäle M, Söderlund T, Koskenvesa P, Talvensaari K, Airaksinen M (2016) Impact of tailored patient education on adherence of patients with chronic myeloid leukaemia to tyrosine kinase inhibitors: a randomized multicentre intervention study. J Adv Nurs 72(9):2196–2206 27113362 10.1111/jan.12978

[41] Tan BK, Chua SS, Chen LC, Chang KM, Balashanker S, Bee PC (2020) Efficacy of a medication management service in improving adherence to tyrosine kinase inhibitors and clinical outcomes of patients with chronic myeloid leukaemia: a randomised controlled trial. Support Care Cancer 28(7):3237–3247 31734798 10.1007/s00520-019-05133-0

[42] Bandiera C, Locatelli I, Courlet P, Cardoso E, Zaman K, Stravodimou A et al (2023) Adherence to the CDK 4/6 inhibitor palbociclib and omission of dose management supported by pharmacometric modelling as part of the OpTAT study. Cancers 15(1):316 36612312 10.3390/cancers15010316 PMC9818079

[43] Khandelwal N, Duncan I, Ahmed T, Rubinstein E, Pegus C (2012) Oral chemotherapy program improves adherence and reduces medication wastage and hospital admissions. J Natl Compr Canc Netw 10(5):618–625 22570292 10.6004/jnccn.2012.0063

[44] Lam MSH, Cheung N (2016) Impact of oncology pharmacist-managed oral anticancer therapy in patients with chronic myelogenous leukemia. J Oncol Pharm Pract 22(6):741–748 26419691 10.1177/1078155215608523

[45] Zerbit J, Chevret S, Bernard S, Kroemer M, Ablard C, Harel S et al (2020) Improved time to treatment failure and survival in ibrutinib-treated malignancies with a pharmaceutical care program: an observational cohort study. Ann Hematol 99(7):1615–1625 32483668 10.1007/s00277-020-04045-y PMC7316844

[46] Dennison T, Deal AM, Foster M, Valgus J, Muluneh B (2021) A pharmacist-led oral chemotherapy program’s impact on chronic myeloid leukemia patient satisfaction, adherence, and outcomes. J Adv Pract Oncol 12(2):148–157 34109047 10.6004/jadpro.2021.12.2.3 PMC8017794

[47] Tan EH, Wong ALA, Tan CC, Wong P, Tan SH, Ang LEY et al (2020) Improving medication adherence with adjuvant aromatase inhibitor in women with breast cancer: a randomised controlled trial to evaluate the effect of short message service (SMS) reminder. Breast 53:77–84 32652462 10.1016/j.breast.2020.06.012 PMC7375684

[48] Ni CX, Lu WJ, Ni M, Huang F, Li DJ, Shen FM (2023) Advanced messaging intervention for medication adherence and clinical outcomes among patients with cancer: randomized controlled trial. JMIR Cancer 9(1):e44612 37651170 10.2196/44612 PMC10502590

[49] McKee M, Frei BL, Garcia A, Fike D, Soefje SA (2011) Impact of clinical pharmacy services on patients in an outpatient chemotherapy academic clinic. J Oncol Pharm Pract 17(4):387–394 21239453 10.1177/1078155210389217

[50] Hurtado-de-Mendoza A, Cabling ML, Lobo T, Dash C, Sheppard VB (2016) Behavioral interventions to enhance adherence to hormone therapy in breast cancer survivors: a systematic literature review. Clin Breast Cancer 16(4):247–255 10.1016/j.clbc.2016.03.006 PMC4969158 27133733

[51] Skrabal Ross X, Gunn KM, Suppiah V, Patterson P, Olver I (2020) A review of factors influencing non-adherence to oral antineoplastic drugs. Support Care Cancer 28:4043–4050 32335731 10.1007/s00520-020-05469-y

[52] Muluneh B, Deal A, Alexander MD, Keisler MD, Markey JM, Neal JM et al (2018) Patient perspectives on the barriers associated with medication adherence to oral chemotherapy. J Oncol Pharm Pract 24(2):98–109 27895220 10.1177/1078155216679026

[53] Geynisman DM, Wickersham KE (2013) Adherence to targeted oral anticancer medications. Discov Med 15(83):231–241 23636140 PMC6477693

[54] Huang WC, Chen CY, Lin SJ, Chang CS (2016) Medication adherence to oral anticancer drugs: systematic review. Expert Rev Anticancer Ther 16(4):423–432 26935964 10.1586/14737140.2016.1159515

[55] Font R, Espinas JA, Gil-Gil M, Barnadas A, Ojeda B, Tusquets I et al (2012) Prescription refill, patient self-report and physician report in assessing adherence to oral endocrine therapy in early breast cancer patients: a retrospective cohort study in Catalonia. Spain Br J Cancer 107(8):1249–1256 22955858 10.1038/bjc.2012.389 PMC3494419

