# Supplementary material for: The effect of healthcare professional-implemented interventions on adherence to oral targeted therapy in patients with cancer: a systematic review and meta-analysis
Source: Support Care Cancer. 2025 Jan 17;33(2):110. doi: 10.1007/s00520-024-09136-4 (PMC11739221; doi:10.1007/s00520-024-09136-4)
Supplement: Supplementary file 1 — Supplementary file1 (DOCX 88 KB) [file 520_2024_9136_MOESM1_ESM.docx]

*Manuscript submitting to Supportive Care in Cancer*

Review

# Title page

**The Effect of Healthcare Professional-Implemented Interventions on Adherence to Oral Targeted Therapy in Patients with Cancer: A Systematic Review and Meta-analysis**

Fiona Angus^1,2^, Wan-Chuen Liao^1,3^, Victoria Adekoya^1^, Li-Chia Chen^1^

1. Centre for Pharmacoepidemiology and Drug Safety, Division of Pharmacy and Optometry, School of Health Sciences, Faculty of Biology, Medicine and Health, University of Manchester, Manchester Academic Health Science Centre, United Kingdom
2. Pharmacy Department, Christie NHS Foundation Trust, Manchester, United Kingdom
3. School of Dentistry, College of Medicine, National Taiwan University, Taipei, Taiwan

**Correspondence to:**

Fiona Angus

Division of Pharmacy and Optometry, School of Health Sciences, Faculty of Biology, Medicine and Health, University of Manchester, Oxford Road, Manchester, M13 9PT, UK.

Email: fiona.angus2@nhs.net

ORCID ID: 0000-0003-0547-1656

# Appendices

Appendix 1. PRISMA 2020 Checklist……………………………………………………3

Appendix 2. Mapping relevant interventions according to the Cochrane Effective Practice and Organisation of Care Taxonomy…………………………………………….5

Appendix 3. Electronic search strategy………………………………………………….7

Appendix 4. Characteristics of the interventions………………………………………21

Appendix 5. The risk-of-bias assessment for randomized controlled trials…………23

Appendix 6. The risk-of-bias assessment for the cohort studies…………………….24

Appendix 1. PRISMA 2020 Checklist

| **Section and Topic** | **Item #** | **Checklist item** | **Location where item is reported** |
| --- | --- | --- | --- |
| **TITLE** | | |  |
| Title | 1 | Identify the report as a systematic review. | 1 |
| **ABSTRACT** | | |  |
| Abstract | 2 | See the PRISMA 2020 for Abstracts checklist. | 2 |
| **INTRODUCTION** | | |  |
| Rationale | 3 | Describe the rationale for the review in the context of existing knowledge. | 3-4 |
| Objectives | 4 | Provide an explicit statement of the objective(s) or question(s) the review addresses. | 3-4 |
| **METHODS** | | |  |
| Eligibility criteria | 5 | Specify the inclusion and exclusion criteria for the review and how studies were grouped for the syntheses. | 5-6, Table 1, Appendix 2-3 |
| Information sources | 6 | Specify all databases, registers, websites, organizations, reference lists and other sources searched or consulted to identify studies. Specify the date when each source was last searched or consulted. | 5-6 |
| Search strategy | 7 | Present the full search strategies for all databases, registers and websites, including any filters and limits used. | 5-6, Appendix 3 |
| Selection process | 8 | Specify the methods used to decide whether a study met the inclusion criteria of the review, including how many reviewers screened each record and each report retrieved, whether they worked independently, and if applicable, details of automation tools used in the process. | 5-7 |
| Data collection process | 9 | Specify the methods used to collect data from reports, including how many reviewers collected data from each report, whether they worked independently, any processes for obtaining or confirming data from study investigators, and if applicable, details of automation tools used in the process. | 5-7 |
| Data items | 10a | List and define all outcomes for which data were sought. Specify whether all results that were compatible with each outcome domain in each study were sought (e.g. for all measures, time points, analyses), and if not, the methods used to decide which results to collect. | 5-7 |
|  | 10b | List and define all other variables for which data were sought (e.g. participant and intervention characteristics, funding sources). Describe any assumptions made about any missing or unclear information. | 5-7 |
| Study risk of bias assessment | 11 | Specify the methods used to assess risk of bias in the included studies, including details of the tool(s) used, how many reviewers assessed each study and whether they worked independently, and if applicable, details of automation tools used in the process. | 5-7 |
| Effect measures | 12 | Specify for each outcome the effect measure(s) (e.g. risk ratio, mean difference) used in the synthesis or presentation of results. | 5-7 |
| Synthesis methods | 13a | Describe the processes used to decide which studies were eligible for each synthesis (e.g. tabulating the study intervention characteristics and comparing against the planned groups for each synthesis (item #5)). | 5-7 |
|  | 13b | Describe any methods required to prepare the data for presentation or synthesis, such as handling of missing summary statistics, or data conversions. | 5-7 |
|  | 13c | Describe any methods used to tabulate or visually display results of individual studies and syntheses. | 5-7 |
|  | 13d | Describe any methods used to synthesize results and provide a rationale for the choice(s). If meta-analysis was performed, describe the model(s), method(s) to identify the presence and extent of statistical heterogeneity, and software package(s) used. | 5-7 |
|  | 13e | Describe any methods used to explore possible causes of heterogeneity among study results (e.g. subgroup analysis, meta-regression). | 5-7 |
|  | 13f | Describe any sensitivity analyses conducted to assess robustness of the synthesized results. | 5-7 |
| Reporting bias assessment | 14 | Describe any methods used to assess risk of bias due to missing results in a synthesis (arising from reporting biases). | 5-7 |
| Certainty assessment | 15 | Describe any methods used to assess certainty (or confidence) in the body of evidence for an outcome. | 5-7 |
| **RESULTS** | | |  |
| Study selection | 16a | Describe the results of the search and selection process, from the number of records identified in the search to the number of studies included in the review, ideally using a flow diagram. | 8, Figure 1 |
|  | 16b | Cite studies that might appear to meet the inclusion criteria, but which were excluded, and explain why they were excluded. | 8 |
| Study characteristics | 17 | Cite each included study and present its characteristics. | 8-9, Table 2, Appendix 4 |
| Risk of bias in studies | 18 | Present assessments of risk of bias for each included study. | 9-10, Appendix 5-6 |
| Results of individual studies | 19 | For all outcomes, present, for each study: (a) summary statistics for each group (where appropriate) and (b) an effect estimate and its precision (e.g. confidence/credible interval), ideally using structured tables or plots. | 8-10 |
| Results of syntheses | 20a | For each synthesis, briefly summarise the characteristics and risk of bias among contributing studies. | 8-10 |
|  | 20b | Present results of all statistical syntheses conducted. If meta-analysis was done, present for each the summary estimate and its precision (e.g. confidence/credible interval) and measures of statistical heterogeneity. If comparing groups, describe the direction of the effect. | 8-10, Table 3-4 |
|  | 20c | Present results of all investigations of possible causes of heterogeneity among study results. | 8-10 |
|  | 20d | Present results of all sensitivity analyses conducted to assess the robustness of the synthesized results. | 8-10 |
| Reporting biases | 21 | Present assessments of risk of bias due to missing results (arising from reporting biases) for each synthesis assessed. | 8-10 |
| Certainty of evidence | 22 | Present assessments of certainty (or confidence) in the body of evidence for each outcome assessed. | 8-10 |
| **DISCUSSION** | | |  |
| Discussion | 23a | Provide a general interpretation of the results in the context of other evidence. | 11 |
|  | 23b | Discuss any limitations of the evidence included in the review. | 11-13 |
|  | 23c | Discuss any limitations of the review processes used. | 11-13 |
|  | 23d | Discuss implications of the results for practice, policy, and future research. | 11-13 |
| **OTHER INFORMATION** | | |  |
| Registration and protocol | 24a | Provide registration information for the review, including register name and registration number, or state that the review was not registered. | 5 |
|  | 24b | Indicate where the review protocol can be accessed, or state that a protocol was not prepared. | 5 |
|  | 24c | Describe and explain any amendments to information provided at registration or in the protocol. | 5 |
| Support | 25 | Describe sources of financial or non-financial support for the review, and the role of the funders or sponsors in the review. | 18 |
| Competing interests | 26 | Declare any competing interests of review authors. | 18 |
| Availability of data, code and other materials | 27 | Report which of the following are publicly available and where they can be found: template data collection forms; data extracted from included studies; data used for all analyses; analytic code; any other materials used in the review. | Not applicable |

*From:*  Page MJ, McKenzie JE, Bossuyt PM, Boutron I, Hoffmann TC, Mulrow CD, et al. The PRISMA 2020 statement: an updated guideline for reporting systematic reviews. BMJ 2021;372:n71. doi: 10.1136/bmj.n71

Appendix 2. Mapping relevant interventions according to the Cochrane Effective Practice and Organisation of Care Taxonomy

| **Subcategory** | **Definition** | **Rationale** |
| --- | --- | --- |
| **1.** **How and when care is delivered** | | |
| Group versus individual care | Comparisons of providing care to groups versus individual patients. | Group or individual care (counselling) of medication use problems and issues related to adherence. |
| Coordination of care amongst different provider | Organizing different providers and services to ensure timely and efficient delivery of healthcare. | See category "Coordination of care and management of care processes." |
| Quality and safety systems | Essential standards for quality of healthcare and reduced poor outcomes related to unsafe healthcare. | Hospital in-house standards are set to ensure optimal adherence in patients with cancer. |
| Triage | Management of patients attending a healthcare facility, contacting a healthcare professional by phone, and receiving advice or being referred to an appropriate service. | Pharmacist-led the triage patients to manage medication use problems and issues related to adherence. |
| **2. Where care is provided and changes to the healthcare environment** | | |
| Outreach services | Visits by health workers to different locations, for example, involving specialists, generalists, or mobile units. | Pharmacists/nurses to do home visits or telephone follow-ups of patients' medication use problems. |
| **3. Who provides care and how the healthcare workforce is managed** | | |
| Role expansion or task shifting. | Expanding tasks undertaken by a cadre of health workers or shifting tasks from one cadre to another to include tasks not previously part of their scope of practice. | This may include substituting one cadre of healthcare work for another.  Pharmacists (or nurses) to expand their roles on leading the management of medication use problems and issues related to adherence. |
| Self-management | Shifting or promoting the responsibility for healthcare or disease management to the patient and/or their family. | Shifting or promoting the responsibility for managing medication taking and adherence to the patient and/or their family. |
| Length of consultation | Changes in the length of consultations. | This is indirect to medication adherence, but it's associated. Extend the consultation length to cover the medication adherence issues. |
| **4. Coordination of care and management of care processes**  Changes in how health workers interact with each other or patients to ensure timely and efficient delivery of healthcare. | | |
| Care pathways | Aim to link evidence to practice for specific health conditions and local arrangements for delivering care. | Link the practice of adherence management into the care pathway. |
| Case management | Introduction, modification or removal of strategies to improve the coordination and continuity of delivery of services, i.e., improving the management of one "case" (patient). | Introduction, modification or removal of strategies to improve the coordination and continuity of interventions to ensure medication adherence. |
| Communication between providers | Systems or strategies for improving communication between healthcare providers, such as systems to improve immunization coverage in LMIC. | Communication between different healthcare professionals to ensure patients' adherence. |
| Continuity of care | Interventions to reduce fragmented care and undesirable consequences of fragmented care, for example, by ensuring the responsibility of care is passed from one facility to another. Hence, the patient perceives their needs and circumstances are known to the provider. | This may not be relevant, but the communication between the hospital setting and the primary care (GP/community pharmacy) is still essential. |
| Discharge planning | An individualized plan of discharge to facilitate the transfer of a patient from the hospital to a post-discharge setting. | This is indirect to medication adherence, but it's associated. |
| Disease management | Programs designed to manage or prevent a chronic condition using a systematic approach to care and potentially employing multiple ways of influencing patients, providers or the process of care. | Mediation management is often included in the disease management plan, which should include strategies to ensure medication adherence. |
| Integration | Consolidating the provision of different healthcare services to one (or simply fewer) facilities. | This may not be relevant, but the communication between the hospital setting and the primary care (GP/community pharmacy) is still essential. |
| Packages of care | Introduction, modification, or removal of packages of services designed to be implemented together for a particular diagnosis/disease, e.g., tuberculosis management guidelines or new-born care protocols. | This is indirect to medication adherence, but it's associated. |
| Patient-initiated appointment systems | Systems that enable patients to make urgent appointments when they feel they cannot manage their condition or where something has changed unexpectedly. | This is indirect to medication adherence, but it's associated. |
| Procurement and distribution of supplies | Systems for procuring and distributing drugs or other supplies. | Ensuring patients have a timely supply of medication is also crucial to ensure adherence. |
| Referral systems | Systems for managing referrals of patients between healthcare providers. | This could be related to the communication between providers and integration care. |
| Shared care | Continuing collaborative clinical care between primary and specialist care physicians. | This could be related to the communication between providers in integration care. |
| Shared decision-making | Sharing healthcare decision-making responsibilities among different individuals, potentially including the patient. | Communication between patients and healthcare professionals to agree on medication taking. |
| Teams | Creating and delivering care through a multidisciplinary team of healthcare workers. | This could be related to the communication between providers and integration care. |
| Transition of Care | Interventions to improve the transition from one care provider to another include adolescents moving from child to adult health services. | This could be related to the communication between providers and integration care. |
| **5. Information and communication technology**  ICT is used by healthcare organizations to manage the delivery of healthcare and to deliver healthcare | | |
| Health information systems | Health record and health management systems to store and manage patient health information, for example, electronic patient records or systems for recalling patients for follow-up or prevention, e.g., immunization. | Follow-up patients' medication use and patient-reported outcomes to identify and prevent suboptimal adherence. |
| The use of information and communication technology | Technology-based methods to transfer healthcare information and support the delivery of care. | Using the information system to transfer information to patients resolves medication use problems and ensures good adherence. |
| Smart home technologies | Electronic assistive technologies. | Using smartphone technology to ensure medication adherence. |
| Telemedicine | Exchange of healthcare information from one site to another via electronic communication. | Telemedicine consultation with patients to ensure medication adherence. |

(Note) LMIC: low-income and middle-income countries. ICT: information and communication technology.

Appendix 3. Electronic search strategy

**MEDLINE**

1. Neoplasms/
2. Cancer.mp.
3. Carcinoma/
4. Tumor.mp.
5. Tumo*.mp.
6. Malignancy.mp.
7. Malignant.mp.
8. Malignan*.mp.
9. 1 or 2 or 3 or 4 or 5 or 6 or 7 or 8
10. Ixazomib.mp.
11. Abemaciclib.mp.
12. Acalabrutinib.mp.
13. Afatinib/
14. Alectinib.mp.
15. Alpelisib.mp.
16. Asciminib.mp.
17. Avapritinib.mp.
18. Axitinib/
19. Binimetinib.mp.
20. Bosutinib.mp.
21. Brigatinib.mp.
22. Cabozantinib.mp.
23. Ceritinib.mp.
24. Cobimetinib.mp.
25. Crizotinib/
26. Dabrafenib.mp.
27. Dacomitinib.mp.
28. Dasatinib/
29. Encorafenib.mp.
30. Entrectinib.mp.
31. Erlotinib.mp.
32. Everolimus/
33. Gefitinib/
34. Gilteritinib.mp.
35. Glasdegib.mp.
36. Ibrutinib.mp.
37. Idelalisib.mp.
38. Imatinib.mp.
39. Lapatinib/
40. Larotrectinib.mp.
41. Lenvatinib.mp.
42. Lorlatinib.mp.
43. Midostaurin.mp.
44. Mobocertinib.mp.
45. Neratinib.mp.
46. Nilotinib.mp.
47. Nintedanib.mp.
48. Niraparib.mp.
49. Olaparib.mp.
50. Osimertinib.mp.
51. Palbociclib.mp.
52. Pazopanib.mp.
53. Pemigatinib.mp.
54. Ponatinib.mp.
55. Pralsetinib.mp.
56. Regorafenib.mp.
57. Ribociclib.mp.
58. Ripretinib.mp.
59. Ruxolitinib.mp.
60. Selpercatinib.mp.
61. Sorafenib/
62. Sotorasib.mp.
63. Sunitinib/
64. Talazoparib.mp.
65. Tepotinib.mp.
66. Tivozanib.mp.
67. Trametinib.mp.
68. Tucatinib.mp.
69. Vandetanib.mp.
70. Vemurafenib/
71. Venetoclax.mp.
72. Vismodegib.mp.
73. Zanubrutinib.mp.
74. 10 or 11 or 12 or 13 or 14 or 15 or 16 or 17 or 18 or 19 or 20 or 21 or 22 or 23 or 24 or 25 or 26 or 27 or 28 or 29 or 30 or 31 or 32 or 33 or 34 or 35 or 36 or 37 or 38 or 39 or 40 or 41 or 42 or 43 or 44 or 45 or 46 or 47 or 48 or 49 or 50 or 51 or 52 or 53 or 54 or 55 or 56 or 57 or 58 or 59 or 60 or 61 or 62 or 63 or 64 or 65 or 66 or 67 or 68 or 69 or 70 or 71 or 72 or 73
75. Behavioural therap*.mp.
76. Cognitive therap*.mp.
77. Educ*.mp.
78. Train*.mp.
79. Teach*.mp.
80. Patient education.mp. or Patient Education as Topic/
81. Counsel*.mp.
82. Consult*.mp.
83. Intervention*.mp.
84. Pharm* intervention.mp.
85. Pharm*-led.mp.
86. Follow up.mp.
87. Cognitive Behavioral Therapy/
88. Psychotherap*.mp.
89. Cognitive psychotherapy*.mp.
90. Cell Phone/
91. Mobile phone.mp.
92. Notification.mp.
93. Reminder.mp.
94. Text Messaging/
95. Mobile text.mp.
96. SMS text.mp
97. Mobile Applications/
98. Mobile app*.mp.
99. Diary.mp.
100. Compliance aids.mp.
101. Adherence aids.mp.
102. 75 or 76 or 77 or 78 or 79 or 80 or 81 or 82 or 83 or 84 or 85 or 86 or 87 or 88 or 89 or 90 or 91 or 92 or 93 or 94 or 95 or 96 or 97 or 98 or 99 or 100 or 101
103. Pharm*.mp.
104. Pharmacists/
105. Healthcare provider.mp.
106. Health provider.mp.
107. Health Personnel/
108. Health professional.mp.
109. Healthcare worker.mp.
110. Physicians/
111. Nurses/
112. Nurs*.mp.
113. Nursing Staff, Hospital/
114. Nursing, Team/
115. Doctor.mp.
116. 103 or 104 or 105 or 106 or 107 or 108 or 109 or 110 or 111 or 112 or 113 or 114 or 115
117. Medication Adherence/
118. Adherence.mp.
119. "Treatment Adherence and Compliance"/
120. Compliance/
121. Persistence.mp.
122. Patient Compliance/
123. Therapy compliance.mp.
124. Drug compliance.mp.
125. Medication persistence.mp.
126. Prescription refill.mp.
127. Medication possess*.mp.
128. Medication gaps.mp.
129. Refill gaps.mp.
130. Medication taking*.mp.
131. 117 or 118 or 119 or 120 or 121 or 122 or 123 or 124 or 125 or 126 or 127 or 128 or 129 or 130
132. 9 and 74 and 102 and 116 and 131
133. limit 132 to (english language and humans)

**EMBASE**

1. Neoplasms/
2. Cancer.mp.
3. Carcinoma/
4. Tumor.mp.
5. Tumo*.mp.
6. Malignancy.mp.
7. Malignant.mp.
8. Malignan*.mp.
9. 1 or 2 or 3 or 4 or 5 or 6 or 7 or 8
10. Ixazomib.mp.
11. Abemaciclib.mp.
12. Acalabrutinib.mp.
13. Afatinib/
14. Alectinib.mp.
15. Alpelisib.mp.
16. Asciminib.mp.
17. Avapritinib.mp.
18. Axitinib/
19. Binimetinib.mp.
20. Bosutinib.mp.
21. Brigatinib.mp.
22. Cabozantinib.mp.
23. Ceritinib.mp.
24. Cobimetinib.mp.
25. Crizotinib/
26. Dabrafenib.mp.
27. Dacomitinib.mp.
28. Dasatinib/
29. Encorafenib.mp.
30. Entrectinib.mp.
31. Erlotinib.mp.
32. Everolimus/
33. Gefitinib/
34. Gilteritinib.mp.
35. Glasdegib.mp.
36. Ibrutinib.mp.
37. Idelalisib.mp.
38. Imatinib.mp.
39. Lapatinib/
40. Larotrectinib.mp.
41. Lenvatinib.mp.
42. Lorlatinib.mp.
43. Midostaurin.mp.
44. Mobocertinib.mp.
45. Neratinib.mp.
46. Nilotinib.mp.
47. Nintedanib.mp.
48. Niraparib.mp.
49. Olaparib.mp.
50. Osimertinib.mp.
51. Palbociclib.mp.
52. Pazopanib.mp.
53. Pemigatinib.mp.
54. Ponatinib.mp.
55. Pralsetinib.mp.
56. Regorafenib.mp.
57. Ribociclib.mp.
58. Ripretinib.mp.
59. Ruxolitinib.mp.
60. Selpercatinib.mp.
61. Sorafenib/
62. Sotorasib.mp.
63. Sunitinib/
64. Talazoparib.mp.
65. Tepotinib.mp.
66. Tivozanib.mp.
67. Trametinib.mp.
68. Tucatinib.mp.
69. Vandetanib.mp.
70. Vemurafenib/
71. Venetoclax.mp.
72. Vismodegib.mp.
73. Zanubrutinib.mp.
74. 10 or 11 or 12 or 13 or 14 or 15 or 16 or 17 or 18 or 19 or 20 or 21 or 22 or 23 or 24 or 25 or 26 or 27 or 28 or 29 or 30 or 31 or 32 or 33 or 34 or 35 or 36 or 37 or 38 or 39 or 40 or 41 or 42 or 43 or 44 or 45 or 46 or 47 or 48 or 49 or 50 or 51 or 52 or 53 or 54 or 55 or 56 or 57 or 58 or 59 or 60 or 61 or 62 or 63 or 64 or 65 or 66 or 67 or 68 or 69 or 70 or 71 or 72 or 73
75. Behavioural therap*.mp.
76. Cognitive therap*.mp.
77. Educ*.mp.
78. Train*.mp.
79. Teach*.mp.
80. Patient education.mp. or Patient Education as Topic/
81. Counsel*.mp.
82. Consult*.mp.
83. Intervention*.mp.
84. Pharm* intervention.mp.
85. Pharm*-led.mp.
86. Follow up.mp.
87. Cognitive Behavioral Therapy/
88. Psychotherap*.mp.
89. Cognitive psychotherapy*.mp.
90. Cell Phone/
91. Mobile phone.mp.
92. Notification.mp.
93. Reminder.mp.
94. Text Messaging/
95. Mobile text.mp.
96. SMS text.mp
97. Mobile Applications/
98. Mobile app*.mp.
99. Diary.mp.
100. Compliance aids.mp.
101. Adherence aids.mp.
102. 75 or 76 or 77 or 78 or 79 or 80 or 81 or 82 or 83 or 84 or 85 or 86 or 87 or 88 or 89 or 90 or 91 or 92 or 93 or 94 or 95 or 96 or 97 or 98 or 99 or 100 or 101
103. Pharm*.mp.
104. Pharmacists/
105. Healthcare provider.mp.
106. Health provider.mp.
107. Health Personnel/
108. Health professional.mp.
109. Healthcare worker.mp.
110. Physicians/
111. Nurses/
112. Nurs*.mp.
113. Nursing Staff, Hospital/
114. Nursing, Team/
115. Doctor.mp.
116. 103 or 104 or 105 or 106 or 107 or 108 or 109 or 110 or 111 or 112 or 113 or 114 or 115
117. Medication Adherence/
118. Adherence.mp.
119. "Treatment Adherence and Compliance"/
120. Compliance/
121. Persistence.mp.
122. Patient Compliance/
123. Therapy compliance.mp.
124. Drug compliance.mp.
125. Medication persistence.mp.
126. Prescription refill.mp.
127. Medication possess*.mp.
128. Medication gaps.mp.
129. Refill gaps.mp.
130. Medication taking*.mp.
131. 117 or 118 or 119 or 120 or 121 or 122 or 123 or 124 or 125 or 126 or 127 or 128 or 129 or 130
132. 9 and 74 and 102 and 116 and 131
133. limit 132 to (english language and humans)

**APA PsycINFO**

1. Neoplasms/
2. Cancer.mp.
3. Carcinoma/
4. Tumor.mp.
5. Tumo*.mp.
6. Malignancy.mp.
7. Malignant.mp.
8. Malignan*.mp.
9. 1 or 2 or 3 or 4 or 5 or 6 or 7 or 8
10. Ixazomib.mp.
11. Abemaciclib.mp.
12. Acalabrutinib.mp.
13. Afatinib/
14. Alectinib.mp.
15. Alpelisib.mp.
16. Asciminib.mp.
17. Avapritinib.mp.
18. Axitinib/
19. Binimetinib.mp.
20. Bosutinib.mp.
21. Brigatinib.mp.
22. Cabozantinib.mp.
23. Ceritinib.mp.
24. Cobimetinib.mp.
25. Crizotinib/
26. Dabrafenib.mp.
27. Dacomitinib.mp.
28. Dasatinib/
29. Encorafenib.mp.
30. Entrectinib.mp.
31. Erlotinib.mp.
32. Everolimus/
33. Gefitinib/
34. Gilteritinib.mp.
35. Glasdegib.mp.
36. Ibrutinib.mp.
37. Idelalisib.mp.
38. Imatinib.mp.
39. Lapatinib/
40. Larotrectinib.mp.
41. Lenvatinib.mp.
42. Lorlatinib.mp.
43. Midostaurin.mp.
44. Mobocertinib.mp.
45. Neratinib.mp.
46. Nilotinib.mp.
47. Nintedanib.mp.
48. Niraparib.mp.
49. Olaparib.mp.
50. Osimertinib.mp.
51. Palbociclib.mp.
52. Pazopanib.mp.
53. Pemigatinib.mp.
54. Ponatinib.mp.
55. Pralsetinib.mp.
56. Regorafenib.mp.
57. Ribociclib.mp.
58. Ripretinib.mp.
59. Ruxolitinib.mp.
60. Selpercatinib.mp.
61. Sorafenib/
62. Sotorasib.mp.
63. Sunitinib/
64. Talazoparib.mp.
65. Tepotinib.mp.
66. Tivozanib.mp.
67. Trametinib.mp.
68. Tucatinib.mp.
69. Vandetanib.mp.
70. Vemurafenib/
71. Venetoclax.mp.
72. Vismodegib.mp.
73. Zanubrutinib.mp.
74. 10 or 11 or 12 or 13 or 14 or 15 or 16 or 17 or 18 or 19 or 20 or 21 or 22 or 23 or 24 or 25 or 26 or 27 or 28 or 29 or 30 or 31 or 32 or 33 or 34 or 35 or 36 or 37 or 38 or 39 or 40 or 41 or 42 or 43 or 44 or 45 or 46 or 47 or 48 or 49 or 50 or 51 or 52 or 53 or 54 or 55 or 56 or 57 or 58 or 59 or 60 or 61 or 62 or 63 or 64 or 65 or 66 or 67 or 68 or 69 or 70 or 71 or 72 or 73
75. Behavioural therap*.mp.
76. Cognitive therap*.mp.
77. Educ*.mp.
78. Train*.mp.
79. Teach*.mp.
80. Patient education.mp. or Patient Education as Topic/
81. Counsel*.mp.
82. Consult*.mp.
83. Intervention*.mp.
84. Pharm* intervention.mp.
85. Pharm*-led.mp.
86. Follow up.mp.
87. Cognitive Behavioral Therapy/
88. Psychotherap*.mp.
89. Cognitive psychotherapy*.mp.
90. Cell Phone/
91. Mobile phone.mp.
92. Notification.mp.
93. Reminder.mp.
94. Text Messaging/
95. Mobile text.mp.
96. SMS text.mp
97. Mobile Applications/
98. Mobile app*.mp.
99. Diary.mp.
100. Compliance aids.mp.
101. Adherence aids.mp.
102. 75 or 76 or 77 or 78 or 79 or 80 or 81 or 82 or 83 or 84 or 85 or 86 or 87 or 88 or 89 or 90 or 91 or 92 or 93 or 94 or 95 or 96 or 97 or 98 or 99 or 100 or 101
103. Pharm*.mp.
104. Pharmacists/
105. Healthcare provider.mp.
106. Health provider.mp.
107. Health Personnel/
108. Health professional.mp.
109. Healthcare worker.mp.
110. Physicians/
111. Nurses/
112. Nurs*.mp.
113. Nursing Staff, Hospital/
114. Nursing, Team/
115. Doctor.mp.
116. 103 or 104 or 105 or 106 or 107 or 108 or 109 or 110 or 111 or 112 or 113 or 114 or 115
117. Medication Adherence/
118. Adherence.mp.
119. "Treatment Adherence and Compliance"/
120. Compliance/
121. Persistence.mp.
122. Patient Compliance/
123. Therapy compliance.mp.
124. Drug compliance.mp.
125. Medication persistence.mp.
126. Prescription refill.mp.
127. Medication possess*.mp.
128. Medication gaps.mp.
129. Refill gaps.mp.
130. Medication taking*.mp.
131. 117 or 118 or 119 or 120 or 121 or 122 or 123 or 124 or 125 or 126 or 127 or 128 or 129 or 130
132. 9 and 74 and 102 and 116 and 131
133. limit 132 to (human and english language)

**CINAHL plus**

(Neoplasms OR Cancer OR Carcinoma OR Tumor OR Tumo* OR Malignancy OR Malignant OR Malignan*) AND

(Ixazomib OR Abemaciclib OR Acalabrutinib OR Afatinib OR Alectinib OR Alpelisib OR Asciminib OR Avapritinib OR Axitinib OR Binimetinib OR Bosutinib OR Brigatinib OR Cabozantinib OR Ceritinib OR Cobimetinib OR Crizotinib OR Dabrafenib OR Dacomitinib OR Dasatinib OR Encorafenib OR Entrectinib OR Erlotinib OR Everolimus OR Gefitinib OR Gilteritinib OR Glasdegib OR Ibrutinib OR Idelalisib OR Imatinib OR Lapatinib OR Larotrectinib OR Lenvatinib OR Lorlatinib OR Midostaurin OR Mobocertinib OR Neratinib OR Nilotinib OR Nintedanib OR Niraparib OR Olaparib OR Osimertinib OR Palbociclib OR Pazopanib OR Pemigatinib OR Ponatinib OR Pralsetinib OR Regorafenib OR Ribociclib OR Ripretinib OR Ruxolitinib OR Selpercatinib OR Sorafenib OR Sotorasib OR Sunitinib OR Talazoparib OR Tepotinib OR Tivozanib OR Trametinib OR Tucatinib OR Vandetanib OR Vemurafenib OR Venetoclax OR Vismodegib OR Zanubrutinib) AND

(Behavioural therap* OR Cognitive therap* OR Educ* OR Train* OR Teach* OR Patient education OR Counsel* OR Consult* OR Intervention* OR Pharm* intervention OR Pharm*-led OR Follow up OR Cognitive Behavioral Therapy OR Psychotherap* OR Cognitive psychotherapy* OR Cell Phone OR Mobile phone OR Notification OR Reminder OR Text Messaging OR Mobile text OR SMS text OR Mobile Applications OR Mobile app* OR Diary OR Compliance aids OR Adherence aids) AND

(Pharm* OR Pharmacists OR Healthcare provider OR Health provider OR Health Personnel OR Health professional OR healthcare worker OR Physicians OR Nurses OR Nurs* OR Nursing Staff OR Nursing OR Doctor) AND

(Medication Adherence OR Adherence OR Treatment Adherence and Compliance OR Compliance OR Persistence OR Patient Compliance OR Therapy compliance OR Drug compliance OR Medication persistence OR Prescription refill OR medication possess* OR Medication gaps OR Refill gaps OR medication taking*)

Limiters

English Language

**PubMed**

(Neoplasms OR Cancer OR Carcinoma OR Tumor OR Tumo* OR Malignancy OR Malignant OR Malignan*) AND

(Ixazomib OR Abemaciclib OR Acalabrutinib OR Afatinib OR Alectinib OR Alpelisib OR Asciminib OR Avapritinib OR Axitinib OR Binimetinib OR Bosutinib OR Brigatinib OR Cabozantinib OR Ceritinib OR Cobimetinib OR Crizotinib OR Dabrafenib OR Dacomitinib OR Dasatinib OR Encorafenib OR Entrectinib OR Erlotinib OR Everolimus OR Gefitinib OR Gilteritinib OR Glasdegib OR Ibrutinib OR Idelalisib OR Imatinib OR Lapatinib OR Larotrectinib OR Lenvatinib OR Lorlatinib OR Midostaurin OR Mobocertinib OR Neratinib OR Nilotinib OR Nintedanib OR Niraparib OR Olaparib OR Osimertinib OR Palbociclib OR Pazopanib OR Pemigatinib OR Ponatinib OR Pralsetinib OR Regorafenib OR Ribociclib OR Ripretinib OR Ruxolitinib OR Selpercatinib OR Sorafenib OR Sotorasib OR Sunitinib OR Talazoparib OR Tepotinib OR Tivozanib OR Trametinib OR Tucatinib OR Vandetanib OR Vemurafenib OR Venetoclax OR Vismodegib OR Zanubrutinib) AND

(Behavioural therap* OR Cognitive therap* OR Educ* OR Train* OR Teach* OR Patient education OR Counsel* OR Consult* OR Intervention* OR Pharm* intervention OR Pharm*-led OR Follow up OR Cognitive Behavioral Therapy OR Psychotherap* OR Cognitive psychotherapy* OR Cell Phone OR Mobile phone OR Notification OR Reminder OR Text Messaging OR Mobile text OR SMS text OR Mobile Applications OR Mobile app* OR Diary OR Compliance aids OR Adherence aids) AND

(Pharm* OR Pharmacists OR Healthcare provider OR Health provider OR Health Personnel OR Health professional OR healthcare worker OR Physicians OR Nurses OR Nurs* OR Nursing Staff OR Nursing OR Doctor) AND

(Medication Adherence OR Adherence OR Treatment Adherence and Compliance OR Compliance OR Persistence OR Patient Compliance OR Therapy compliance OR Drug compliance OR Medication persistence OR Prescription refill OR medication possess* OR Medication gaps OR Refill gaps OR medication taking*)

Filters applied: Humans, English.

**Web of Science**

(Neoplasms OR Cancer OR Carcinoma OR Tumor OR Tumo* OR Malignancy OR Malignant OR Malignan*) AND

(Ixazomib OR Abemaciclib OR Acalabrutinib OR Afatinib OR Alectinib OR Alpelisib OR Asciminib OR Avapritinib OR Axitinib OR Binimetinib OR Bosutinib OR Brigatinib OR Cabozantinib OR Ceritinib OR Cobimetinib OR Crizotinib OR Dabrafenib OR Dacomitinib OR Dasatinib OR Encorafenib OR Entrectinib OR Erlotinib OR Everolimus OR Gefitinib OR Gilteritinib OR Glasdegib OR Ibrutinib OR Idelalisib OR Imatinib OR Lapatinib OR Larotrectinib OR Lenvatinib OR Lorlatinib OR Midostaurin OR Mobocertinib OR Neratinib OR Nilotinib OR Nintedanib OR Niraparib OR Olaparib OR Osimertinib OR Palbociclib OR Pazopanib OR Pemigatinib OR Ponatinib OR Pralsetinib OR Regorafenib OR Ribociclib OR Ripretinib OR Ruxolitinib OR Selpercatinib OR Sorafenib OR Sotorasib OR Sunitinib OR Talazoparib OR Tepotinib OR Tivozanib OR Trametinib OR Tucatinib OR Vandetanib OR Vemurafenib OR Venetoclax OR Vismodegib OR Zanubrutinib) AND

(Behavioural therap* OR Cognitive therap* OR Educ* OR Train* OR Teach* OR Patient education OR Counsel* OR Consult* OR Intervention* OR Pharm* intervention OR Pharm*-led OR Follow up OR Cognitive Behavioral Therapy OR Psychotherap* OR Cognitive psychotherapy* OR Cell Phone OR Mobile phone OR Notification OR Reminder OR Text Messaging OR Mobile text OR SMS text OR Mobile Applications OR Mobile app* OR Diary OR Compliance aids OR Adherence aids) AND

(Pharm* OR Pharmacists OR Healthcare provider OR Health provider OR Health Personnel OR Health professional OR healthcare worker OR Physicians OR Nurses OR Nurs* OR Nursing Staff OR Nursing OR Doctor) AND

(Medication Adherence OR Adherence OR Treatment Adherence and Compliance OR Compliance OR Persistence OR Patient Compliance OR Therapy compliance OR Drug compliance OR Medication persistence OR Prescription refill OR medication possess* OR Medication gaps OR Refill gaps OR medication taking*)

Language: English

Appendix 4. Characteristics of the interventions

| **Author, year, country** | **Settings** | **Medication delivery** | **Healthcare professional** | **Key contents** | | | | **Strategy to suboptimal** | **Coordination** | **Delivery mode** | **Intensity** | **Duration** |
| --- | --- | --- | --- | --- | --- | --- | --- | --- | --- | --- | --- | --- |
|  |  |  |  | **Disease and medication education** | **Side effect management** | **Adherence monitoring** | **Drug interaction review** |  |  |  |  |  |
| Khandelwal, 2012, US | Specialty pharmacy | Patient self-administered | Oncology nurses and pharmacists | V | V | V |  | Not specified | The physician is notified of adherence issues and side effects | Telephone | Patients are contacted on days 10 and 20 of the first month, then monthly, with 24/7 access to a pharmacist via a toll-free number. | 6 months |
| Kajizono, 2015, Japan | University hospital | Patient self-administered | Two pharmacists (including one oncology pharmacist) and one nurse |  | V |  |  | Not specified | Physicians are provided with recommendations | Face-to-face | Once every month with a median intervention time of 40 minutes | 43 months |
| Kekale, 2016, Finland | Secondary and tertiary care hospitals | Patient self-administered | Hematology nurses | V | V | V |  | Daily texts for medication reminders | Not specified | Face-to-face and multimedia | 30-minute counselling, a 5-minute video, and daily text messages | 9 months |
| Lam, 2016, US | Oncology clinics | Patient self-administered | Oncology pharmacists | V | V | V | V | Not specified | Not specified | Face-to-face | Weekly to every 3-month | 6 years |
| Todo, 2019, Japan | Outpatient cancer chemotherapy clinic | Patient self-administered | Pharmacists (including one oncology pharmacist) | V | V | V |  | Not specified | Interview information was shared with healthcare professionals, and physicians are advised on relieving adverse events. | Face-to-face | Interviews were conducted at every patient visit, but the frequency was not specified. | 26 months |
| Tan, 2020, Malaysia | Hospitals | Patient self-administered | Research pharmacist | V | V | V | V | Medication packaging was marked with calendar dates, and the MediSafe application was used for reminders. | Not specified | Three face-to-face reviews, two follow-up calls, a booklet, and two adherence aids. | Three face-to-face study visits on the 1st, 3rd, and 6th months after recruitment. | 6 months |
| Zerbit, 2020, France | Hospital | Patient self-administered | Clinical pharmacists | V | V | V | V | Possible causes were addressed, and familiar caregivers were trained. | Physicians were advised on drug-drug interactions. | Face-to-face | Patients were seen every 3 months for the first 6 months and then every 6 months, with at least four 30- to 60-minute pharmaceutical consultations. | Median (IQR): 13.8 (7.6, 19.8) months |
| Dennison, 2021, US | University medical center | Patient self-administered | Oncology clinical pharmacist | V | V | V | V | Visits will be individualized every 2-4 weeks as needed. | Not specified | Face-to-face | 1-2 weeks, 4-6 weeks, and 3 months post medication initiation. | At least 3 months |
| Hirabatake, 2023, Japan | Medical center general hospital | Patient self-administered | Oncology pharmacists |  | V | V | V | Not specified | Urologists were given evaluation findings and suggestions. | Face-to-face | 15-min face-to-face consultation, (median [IQR] 8 times [4–19 times]) | 51 months |
| Bandiera, 2023, Switzerland | University hospital | Delivered in electronic monitors | Pharmacists | V | V | V |  | Not specified | Intervention reports were provided to oncologists and healthcare professionals. | Face-to-face | Monthly 15-20 min medication adherence interview | 12 months |
| Bandiera, 2024, Switzerland | University hospital | Delivered in electronic monitors. | Pharmacists | V | V | V |  | Explored the patient's willingness and capacity to change their behavior. | Adherence report will be summarized to the healthcare team. | Face-to-face | Interview for 15 minutes every month | 12 months |

(note) US: United States. IQR: interquartile range.

Appendix 5. The risk-of-bias assessment for randomized controlled trials

| **Author, year** | **Bias arising from the randomization process** | **Bias due to deviations from intended intervention** | **Bias due to missing outcome data** | **Bias in the measurement of the outcome** | **Bias in the selection of the reported result** | **Overall** |
| --- | --- | --- | --- | --- | --- | --- |
| Kekale, 2016 | Some concerns | Low risk | Some concerns | Some concerns | Low risk | Some concerns |
| Tan, 2020 | Some concerns | Low risk | Low risk | Low risk | Some concerns | Some concerns |
| Bandiera, 2023 | Some concerns | Low risk | Low risk | Low risk | Some concerns | Some concerns |
| Bandiera, 2024 | Some concerns | Low risk | Some concerns | Low risk | Low risk | Some concerns |

(Note) The risk-of-bias tool for randomized trials (RoB 2) was applied. Background color caption: green: low risk of bias; yellow: some concerns; and red: high risk of bias.

Appendix 6. The risk-of-bias assessment for the cohort studies

| **Author, year** | **Bias due to confounding** | **Bias due to the selection of participants** | **Bias in the classification of interventions** | **Bias due to deviations from intended interventions** | **Bias due to missing data** | **Bias in the measurement of outcomes** | **Bias in the selection of the reported result** | **Overall** |
| --- | --- | --- | --- | --- | --- | --- | --- | --- |
| Khandelwal, 2012 | Moderate | Low | Low | Low | Low | Low | Moderate | Moderate |
| Kajizono, 2015 | Moderate | Low | Low | Low | Low | Low | Low | Moderate |
| Lam, 2016 | Moderate | Low | Low | Low | Low | Low | Moderate | Moderate |
| Todo, 2019 | Moderate | Low | Low | Low | Low | Low | Low | Moderate |
| Zerbit, 2020 | Moderate | Low | Low | Low | Low | Low | Low | Moderate |
| Dennison, 2021 | Moderate | Low | Low | Low | Low | Moderate | Moderate | Moderate |
| Hirabatake, 2023 | Moderate | Low | Low | Low | Low | Low | Low | Moderate |

(Note) The risk of bias in non-randomized studies of interventions (ROBINS-I) tool was applied. Background color caption: green; low risk of bias (the study is comparable to a well-performed randomized trial concerning this domain); yellow: moderate risk of bias (the study is sound for a non-randomized study concerning this domain but cannot be considered comparable to a well-performed randomized trial); orange: serious risk of bias (the study has some important problems); and red critical risk of bias (the study is too problematic to provide any useful evidence on the effects of intervention).
